# Expression of p-AMPK in colorectal cancer revealed substantial diverse survival patterns

**DOI:** 10.12669/pjms.35.3.159

**Published:** 2019

**Authors:** Mohamad Nidal Khabaz, Amer Shafei Abdelrahman, Jaudah Ahmad Al-Maghrabi

**Affiliations:** 1 *Prof. Mohamad Nidal Khabaz, MD, PhD. Rabigh Faculty of Medicine, Department of Pathology, King Abdulaziz University Jeddah, Saudi Arabia*; 2 *Dr. Amer Shafei Abdelrahman, MD, PhD. Rabigh Faculty of Medicine, Department of Pathology, King Abdulaziz University Jeddah, Saudi Arabia*; 3 *Prof. Jaudah Ahmad Al-Maghrabi, FRCPC. Faculty of Medicine, Department of Pathology, King Abdulaziz University Jeddah, Saudi Arabia*

**Keywords:** Phosphorylated AMPK, p-AMPK, Immunohistochemistry, Colorectal cancer

## Abstract

**Objective:**

Several cancers have showed differences in the role of p- AMPK in cancer growth, progression and prognosis, and little is identified regarding the significance of p-AMPK expression in colorectal adenocarcinoma. Therefore, this report will define p-AMPK phenotype in a panel of colorectal carcinomas and explore the relationship between this phenotype and tumor clinicopathological features.

**Methods:**

A total of 228 cases comprising 155 large intestine cancers and 73 controls (40 benign tumors and thirty three non-cancerous tissues) were employed in tissue microarray construction. Immunohistochemistry (IHC) staining was applied to reveal p-AMPK expression. This study was carried out in the pathology lab of King Abdulaziz University Hospital over a duration of 15 months and was completed on 7^th^ July 2018.

**Results:**

Phosphorylated AMPK was identified in 133 (85.8%) of colorectal cancers and 73 (100%) control cases. Histologic type was noticeably correlated with p-AMPK immunostaining (P= 0.001), high score of p-AMPK immunostaining is more frequent in control cases. Considerable varied survival models were observed with neoplasm size, metastatic tumor, recurrence and disease relapse (P-values<0.01). Survival estimates are considerably healthier in positive cases which have one of the following features size less than 5 cm, absence of metastatic tumor, no reoccurrence or disease relapse.

**Conclusions:**

The present study showed a reduction in the IHC staining of p-AMPK in colorectal cancer compared with controls. IHC staining of p-AMPK can be a supportive marker in predicting prognosis and survival estimates of colorectal tumors with specific clinical factors.

## INTRODUCTION

Colorectal neoplasms are important cause of mortality in Saudi Arabia. Almost 1400 colorectal tumors were registered in 2013, which counted about 11.9% of all new diagnosed neoplasms.^1^ The most frequent histotype was adenocarcinoma (not otherwise specified) and mucinous carcinoma was far less, then signet ring cell carcinoma, and other types. Colorectal cancer patients who are less than 50 years of age counted for 19.9%.^1^

Colorectal tumor treatment is determined based on patients’ clinical factors, such as grade and stage, infiltration and lymph nodes involvement.^2^ Nonetheless, these data are not sufficient to predict clinical outcomes and a large discrepancies are observed in the consequences especially in cases with equal stage. Thus, other predictive markers at the molecular and histopathological levels are main concern to distinguish and permit selection of cases with severe outcomes.^2^ AMP-activated protein kinase (AMPK) is a cellular energy sensor that can be activated by adding phosphate group as a consequence to metabolic stress that depletes ATP (contraction of muscle) or prevents ATP assembly (glucose deficiency, ischemia and hypoxia) and so increases the AMP:ATP ratio.^3^ It is known that AMPK inhibits mostly all anabolic activities which help cells proliferation and consequently inhibits tumor growth and development.^4^ Once motivated, p-AMPK controls energy-diminishing processes like cellular proliferation and stimulates energy producing reaction like lipid oxidation, glucose uptake and glycolysis. Other anti-tumor potentials of AMPK is growing DNA repair and autophagy following ultraviolet damage through activation and phosphorylation of p21 and p53; consequently distracts cell cycle and promotes cells survival. AMPK has been realized to restrain mTOR and reduces synthesis of proteins.^4^ Moreover, phosphorylated AMPK, in neoplastic cells, inhibits enzymes of lipogenesis which were found in tumor compartment because of increased demands to incorporate fatty acids in the cytoplasmic membrane of proliferating cells.^3^-^5^ There are numerous reports which had described the functions of activated AMPK in tumor growth and progression.^3^,^4^,^6^-^8^ Some of these studies have allied phosphorylated AMPK with good prognosis in several types of tumors including stomach^9^, head and neck^10^, kidney^11^, lung^12^, liver^13^ and breast.^14^ So far, little is identified about the significant role of phosphorylated AMPK (p-AMPK) immunophenotype in colorectal cancer. Therefore, this research will describe p-AMPK phenotype in colorectal carcinomas using immunohistochemistry (IHC) method, and will investigate the relationship between p-AMPK immunoexpression and clinicopathological features.

## METHODS

All tissue samples of colorectal cancer cases which was employed in this research (155 cases: 72 females and 83 males) were obtained from the Department of Pathology at King Abdulaziz University as specimens embedded in paraffin wax as well as control group that includes specimens of normal tissues, non-cancerous conditions, adenomas and adjacent tissues. Paraffin embedded specimens were cut into four µm slides, stained by hematoxylin and eosin and reevaluated. Neoplasm clinicopathological data were gathered from the medical records unit (Table-I). All paraffin embedded tissue specimens of controls and colorectal tumors were utilized to build tissue microarray (TMA). King Abdulaziz University’s Ethical Unit approved the present research. This study was carried out in the pathology lab of King Abdulaziz University Hospital over duration of 15 months and was completed on 7^th^ July 2018.

**Table-I:** Distribution of clinicopathological variables with p-AMPK immunostaining in colorectal adenocarcinomas.

		p-AMPK Immunostaining						P-Value

		Negative		Low		High		

		Count	Row N %	Count	Row N %	Count	Row N %	
Tissue type	Colon adenomas	0	0.0%	7	17.5%	33	82.5%	0.001^a^
	Colorectal Cancer	22	14.2%	45	29.0%	88	56.8%	
	Non-Cancerous	0	0.0%	7	21.2%	26	78.8%	
Age	=<60	10	11.1%	22	26.2%	52	61.9%	0.361^b^
	>60	12	16.9%	23	32.4%	36	50.7%	
Sex	Male	14	16.9%	25	30.1%	44	53.0%	0.492^b^
	Female	8	11.1%	20	27.8%	44	61.1%	
Tumor Differentiation	Well Diff	6	17.1%	9	25.7%	20	57.1%	0.184^a^
	Mod Diff	16	15.7%	27	26.5%	59	57.8%	
	Poorly Diff	0	0.0%	9	50.0%	9	50.0%	
Tumor Location	Right colon	5	12.2%	17	41.5%	19	46.3%	0.075^a^
	Left Colon	12	12.4%	23	23.7%	62	63.9%	
	Rectum	5	29.4%	5	29.4%	7	41.2%	
Tumor Size	< 5cm	12	17.9%	15	22.4%	40	59.7%	0.196^b^
	≥ 5cm	10	11.4%	30	34.1%	48	54.5%	
Tumor Stage	1	1	50.0%	0	0.0%	1	50.0%	0.830^a^
	2	4	13.8%	8	27.6%	17	58.6%	
	3	16	14.0%	33	28.9%	65	57.0%	
	4	1	10.0%	4	40.0%	5	50.0%	
Lymphovascular Invasion	Negative	22	16.4%	37	27.6%	75	56.0%	0.099^a^
	Positive	0	0.0%	8	38.1%	13	61.9%	
Serosa Resected Margin	Negative	21	14.2%	41	27.7%	86	58.1%	0.231^a^
	Positive	1	14.3%	4	57.1%	2	28.6%	
Lymph Node Metastasis	Negative	15	16.3%	28	30.4%	49	53.3%	0.508^a^
	Positive	7	11.1%	17	27.0%	39	61.9%	
Distant Metastasis	Negative	13	11.5%	34	30.1%	66	58.4%	0.300^b^
	Positive	9	21.4%	11	26.2%	22	52.4%	
Local Recurrence	Negative	15	14.0%	34	31.8%	58	54.2%	0.540^b^
	Positive	7	14.6%	11	22.9%	30	62.5%	
Tumor Relapse	Negative	13	13.1%	31	31.3%	55	55.6%	0.709^b^
	Positive	9	16.1%	14	25.0%	33	58.9%	

a Fisher’s exact test;

b Chi-Square test

### Tissue Microarray Construction

TMA was made as it has been illustrated in our earlier papers.^15^ All recruited tumor cases and control samples were utilized in tissue microarray assembly. Tissue microarray blocks were cut 4 µm slices and were placed on aminosilane coated slides, then they were used in IHC staining.

### Immunohistochemistry

Immunohistochemistry staining was accomplished by employing Benchmark ULTRA IHC autostainer (Ventana, Arizona, USA) as we reported previously.^15^ Anti-p-AMPK antibodies (Santa Cruz Biotechnology, USA) were diluted to a ratio of 1 to 100 and applied on sections, next dab visualizing system were added. A negative control slide that contains tris buffer instead of primary antibody were added to every staining run as well as positive control slide of Hep G2 cell lysate which was bought with the primary antibody (Santa Cruz Biotechnology, USA) was included. Every case showed brown staining in greater than 5 percent of neoplastic cells were considered positively stained.

The estimates of p-AMPK positive cells was calculated by semi quantitative technique in three fields using forty amplification power lenses. Scores of zero, one, two, and three were given for negative, faint, moderate and strong staining respectively. These scores are displayed in this report as high (2 and 3), low (1) and negative. Two pathologists scored the intensity of p-AMPK immunostaining and estimated the percentage of positive tumor cells. The smallest scoring value of the two pathologists was considered if a disparity among them was occurred.

### Statistical Analysis

The data were analysed by using version 21 of IBM-SPSS. The relationship between clinical factors and p-AMPK immunoexpression was investigated by Fisher and chi-square tests in Table-I. Assessment of survival distributions for several p-AMPK IHC staining scores were calculated by using Log Rank test. Significance level was considered at *P* < 0.05.

## RESULTS

**Fig.1:**
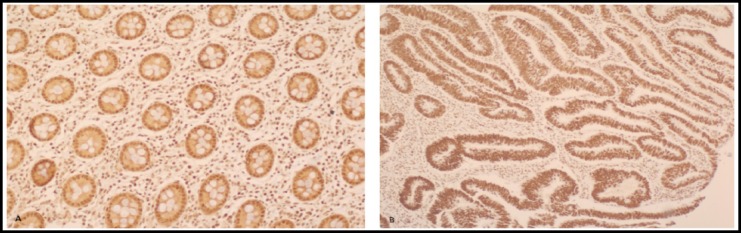
A, strong positive p-AMPK staining in colorectal tissue (10 X); B, strong positive p-AMPK staining in colorectal cancer (10 X).

Clinicopathological factors of all cancer cases with the expression of phosphorylated AMPK is presented in Table-I. 85.8% of tumors presented positive p-AMPK staining, of which 56.8% samples revealed modest to strong staining. More than 94% of positive cases showed nuclear expression of p-AMPK, while the remaining cases revealed nuclear and cytoplasmic locations. Most of positive cases showed brown color in greater than 65% of the transformed cells. All control tissues were p-AMPK positive, of which 78.8% displayed moderate to strong staining (Fig.1 A, B).

Substantial heterogeneity was identified in p-AMPK stain, for instance, some neoplasms exhibited positive stain in selected glands or cells and others showed identical stain in all glandular or cellular parts. P-AMPK expression in colorectal adenocarcinomas showed significant variation from control group (p=0.001), higher proportion of strong immunostaining is observed in colon adenomas and non-cancerous cases. Male and female cancer cases showed nearly similar distribution patterns of p-AMPK expression with small rise in stain intensity of female cases. No considerable relationships were realized between p-AMPK immunostaining and sex, age, size, differentiation, stage, neoplasm location, margins involvement, vascular infiltration, lymph node involvement, metastatic tumors, reoccurrence and relapse of disease (Table-I).

Log Rank test was used to compare survival distributions among colorectal neoplasm cases of low and high phosphorylated AMPK staining scores. Table-II defines the average survival times of tumor patients with different clinical risk factors varied for p-AMPK stain. Substantial diverse survival patterns were detected with size of tumor, metastatic tumors, reoccurrence and relapse of disease. Positively stained tumor of size less than 5cm exhibits better survival time than large neoplasms (P-values 0.016). Positive immunostaining tumors with no metastasis, no reoccurrence or no relapse shows significant improved survival experience (P-values <0.01).

**Table-II:** Comparison of survival distribution patterns by various clinicopathological variables in positive p-AMPK immunostained colorectal adenocarcinomas.

		Mean Survival Time in Months				P-Value^a^

		Estimate	Std. Error	95% Confidence Interval		

				Lower Bound	Upper Bound	
Tumor Size	< 5cm	132.092	7.884	116.639	147.545	0.016
	≥ 5cm	88.432	13.022	62.909	113.955	
Distant Metastasis	Negative	125.187	11.284	103.071	147.304	0.000
	Positive	51.236	7.978	35.599	66.874	
Local Recurrence	Negative	126.407	11.510	103.847	148.967	0.001
	Positive	59.621	8.544	42.874	76.367	
Tumor Relapse	Negative	131.277	11.813	108.124	154.431	0.000
	Positive	59.070	8.014	43.363	74.778	

a Log-Rank test adjusted for p-AMPK Immunostaining

**Fig.2:**
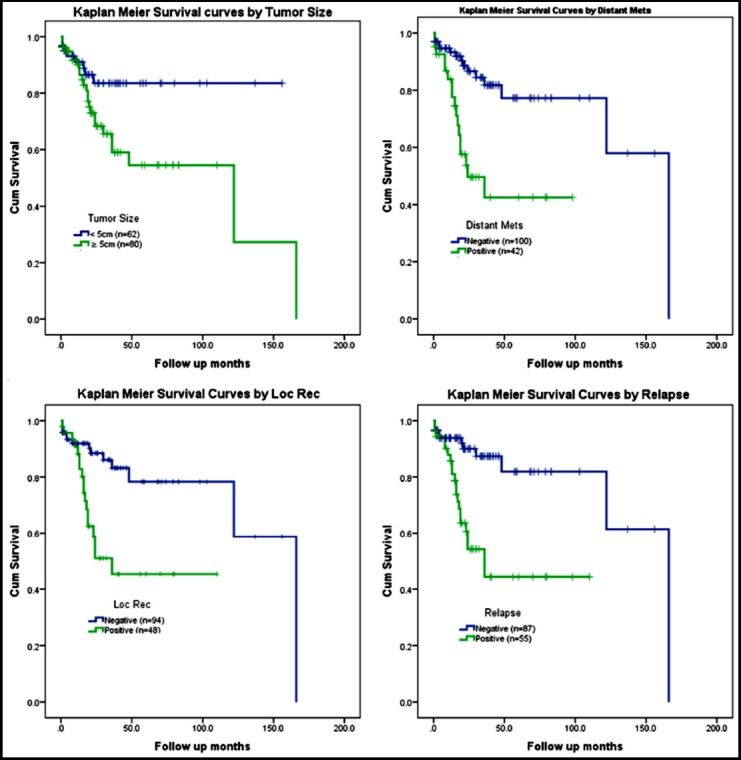
Kaplan Meier Survival Curves by various clinicopathological variables with p-AMPK immunostaining in colorectal adenocarcinoma. Loc Rec: Local Recurrence

On the other hand, positive neoplasms with metastases, reoccurrence or relapse displayed poorer survival estimations. Kaplan Meier survival curves exhibited significant improved survival experience in small neoplasms (less than 5cm) and tumors with no metastases, no reoccurrence or relapse (Fig.2).

## DISCUSSION

There are only two studies, to the best of our knowledge, which assessed p-AMPK expression patterns in colorectal carcinomas.^16^,^17^ Baba and colleagues^16^ found phosphorylated AMPK expression in 409 tumors (57%) of the total 718 colorectal carcinomas by IHC. P-AMPK was allied inversely with high grade tumors (*P*=0.0009). Because they did not use the same clinicopathological factors of the present study, they also found that phosphorylated AMPK phenotype was not considerably correlated with colorectal tumor survival time using Kaplan Meier test. Furthermore, Baba and colleagues study^16^ did not find any impact of phosphorylated AMPK phenotype on the survival estimates of colorectal cancer in relation to clinicopathological factors. Zulato et al.^17^ reported phosphorylated AMPK expression was found in the cytoplasm of transformed cells of thirty-four patients (70.8%) only, and was not associated with clinical data and survival estimates (*P*=0.23).

Both studies did not use control group to compare the expression level of p-AMPK between colorectal cancer and non-cancerous tissues, while the present study reached to a conclusion that there is a reduction in the immunoexpression of p-AMPK in colorectal tumors. In the present study, the high level of p-AMPK expression in colorectal cancer is consistent with Baba et al.^16^ findings, and differed slightly from Zulato et al.^17^ results. Similarly the AMPK phosphorylation activity was described to be suppressed in several tumors such as hepatocellular carcinoma, gastric cancer and breast cancer.^9^,^13^,^14^ Suppressed activation of AMPK may induce tumor growth through deactivation of a tumor suppressing axis LKB1/AMPK signaling route.^18^ Phosphorylated AMPK can efficiently stop the mTOR pathway which is commonly stimulated in several tumors.^19^ Thus, reduced activation of AMPK, a frequent outcome in cancers, decreased the capacity of preventing mTOR pathway.^20^ Otherwise, the suppressed p-AMPK expression in various tumors including colorectal cancer can be accredited partially to a decreased AMPK activation which could be due to decreased amount of total AMPK protein.

Although all studies that described the expression phenotype of p-AMPK in colorectal cancer including the present report found no statistically significant association with clinicopathological parameters except Baba et al.^16^ who reported inverse association with high tumor grade only. The present investigation showed that the impact of p-AMPK phenotype on the survival estimates of colorectal cancer was modified significantly by some clinical factors including size of tumor, metastatic tumors, reoccurrence and disease relapse. This prognostic value of p-AMPK in colorectal tumors is in agreement with other studies of several tumors 9,14 but not the studies of Baba et al.^16^ and Zulato et al.^17^ which reported no important modifying influence by any variable.

In the current analysis, the notable p-AMPK expression in tumor cases might be of clinical importance and may help understand the function of phosphorylated AMPK in colorectal cancer development and tumor cell survival. Moreover, it suggests that, in certain environments, the commonly recognized function of phosphorylated AMPK as a cancer suppressing molecule might be weakened by tumor cells through appropriating p-AMPK to stimulate metabolic modifications to support cell proliferation and survival.^21^ Otherwise, AMPK could work like a binary protein in cancer progression and development based on some factors such as AMPK isoform, level of activation and other motivated compensatory cellular activities. It is possible that medium AMPK phosphorylation that is stimulated by modest stress might apply protective powers and make an oncogenic-like activities, while severe stress might motivate AMPK to show suppressing behaviors and initiate neoplastic cell death.

## CONCLUSION

Our results showed decreased expression of phosphorylated AMPK in colorectal tumors contrasted with controls. IHC staining of activated AMPK can be a supportive mean in predicting prognosis and survival estimates of colorectal tumors with specific clinical factors.
